# Effects of using conventional assistive devices on spatiotemporal gait parameters of adults with neurological disorders: A systematic review protocol

**DOI:** 10.1371/journal.pone.0321019

**Published:** 2025-04-11

**Authors:** Jordana de Paula Magalhães, Sheridan Ayessa Ferreira de Brito, Merrill Landers, Aline Alvim Scianni, Poliana do Amaral Yamaguchi Benfica, Carolina Luisa de Almeida Soares, Christina Danielli Coelho de Morais Faria

**Affiliations:** 1 Department of Physical Therapy, Universidade Federal de Minas Gerais, Belo Horizonte, Brazil; 2 Department of Physical Therapy, Universidade Federal de Minas Gerais, Belo Horizonte, Brazil; 3 Department of Physical Therapy, University of Nevada, Las Vegas, Nevada, United States of America; 4 Department of Physical Therapy, Universidade Federal de Minas Gerais, Belo Horizonte, Brazil; 5 Department of Physical Therapy, Universidade Federal de Minas Gerais, Belo Horizonte, Brazil; 6 Department of Physical Therapy, Universidade Federal de Minas Gerais, Belo Horizonte, Brazil; 7 Department of Physical Therapy, Universidade Federal de Minas Gerais, Belo Horizonte, Brazil; Pennsylvania State University, UNITED STATES OF AMERICA

## Abstract

Individuals with neurological disorders often experience gait impairments that contribute to increased disability, long-term care risk, and higher healthcare costs. The prescription of assistive devices is a commonly employed strategy to compensate for gait impairments in this population. Despite being recommended by various guidelines, there are limited recommendations for prescribing these devices. Furthermore, the effects of using assistive devices on the gait of individuals with neurological disorders are poorly known. Therefore, the aim of this systematic review is to investigate the immediate, short, and long-term effects of using conventional assistive devices (e.g., canes, crutches, walkers) on gait parameters of adults with neurological disorders. This Systematic review was registered in the International Prospective Register of Systematic Reviews (PROSPERO) (registration number: CRD42024542695) and was conducted following Preferred Reporting Items for Systematic Review and Meta-Analysis Protocols (PRISMA-P). Experimental studies that investigated the effects of using conventional assistive devices on the gait of individuals with neurological disorders will be included. Electronic searches will be conducted in Medical Literature Analysis and Retrieval System Online (MEDLINE Ovid), Excerpta Medica Database (Embase Classic +  Embase Ovid), Physiotherapy Evidence Database (PEDro), Scientific Electronic Library Online (Scielo), Cumulative Index to Nursing and Allied Health Literature (CINAHL Database), Literatura Latino-Americana e do Caribe em Ciências da Saúde (LILACS) and gray literature. The reference lists of the included studies will be manually searched. Two trained independent reviewers will select the studies, extract the data, and assess the methodological quality of the included studies using the Cochrane Risk of Bias tools. Disagreements between reviewers will be solved through consensus or by a third independent reviewer. The quality of the evidence will be assessed (GRADE). If a sufficient number of comparable studies are available, subgroup analysis will be conducted and we will consider doing a meta-analysis if the studies are homogeneous. The review will be reported following the Preferred Reporting Items for Systematic Review and Meta-Analysis (PRISMA 2020 statement). The results of this review will provide useful information about the effects of assistive devices on gait in different neurological disorders comprehensively and systematically. Investigating the immediate, short and long-term effects may generate useful information for making clinical decisions related to the training time for using these devices. If a meta-analysis is possible, the prescription of these devices could be improved based on knowledge of their effects. Finally, the results of this systematic review will identify gaps in the literature and guide future research.

## Introduction

Neurological disorders are the leading cause of disability-adjusted life-year (DALYs, sum of years of lives lost and years lived with disability) worldwide [1]. Between 1990 and 2016, there was a 15% rise in DALYs associated with neurological disorders [1]. Only in 2019, neurological disorders (e.g., stroke, Alzheimer’s disease, Parkinson’s disease, multiple sclerosis, brain and spine injuries) were responsible for more than 180 thousand DALYs globally [2]. Therefore, the burden associated with disabilities secondary to neurological disorders is increasingly recognized as a global public health challenge [3]. Furthermore, this burden is expected to increase in the coming decades as a result of the population aging [3].

Individuals with neurological disorders commonly have gait impairments that increase disability and the risk of falls, as well as favoring the adoption of a sedentary lifestyle in this population [4]. In addition, gait impairments are also associated with decreased social participation, and increased risk of long-term care and healthcare costs [5]. Conventionally, gait impairments are quantified by parameters such as gait speed, cadence, and step length [4,6]. These parameters have been useful for evaluating functionality and health, as they can predict various outcomes, including response to rehabilitation, level of functional dependence, mobility limitations, cognitive decline, risk of falls, quality of life, frailty, and mortality [7,8]. Gait speed, for instance, is considered the “sixth vital sign”, as it represents a simple, valid, reliable, sensitive measure capable of assessing and monitoring the functional status of different populations, including individuals with neurological disorders [4,8]. Therefore, several pharmacological and non-pharmacological interventions have been investigated with the aim of improving different gait parameters for diverse health conditions.

The prescription of assistive devices such as canes, crutches, and walkers is a common strategy to compensate for gait impairments [9]. Assistive devices increase the base of support around the center of gravity, improving balance and decreasing walking effort [10]. Clinical guidelines for different neurological disorders recommend the use of different assistive devices as a strategy to increase independence and safety during gait [10–13]. In general, the recommendations for prescribing these devices are based on factors such as the level of external support required and the individual’s ability to manage the device [14]. However, although these recommendations help rehabilitation professionals prescribe devices, they are insufficient to address specific therapeutic goals, such as improving gait speed or capacity.

Studies have shown that around 50% of individuals after stroke use some assistive device, with canes being the most used [10,15]. Likewise, in individuals with spinal cord injury, these devices are also commonly used and the prescription of less restrictive devices, such as canes and walkers, is recommended whenever possible [16]. In individuals with Parkinson’s Disease (PD), the use of assistive devices and the perception of the need for use increases with disease progression [17].

Although being widely used and recommended for individuals with different neurological disorders, recommendations for the prescription of assistive devices are limited. For example, clinical guidelines do not describe the effects of using different devices on gait parameters, such as walking speed—a key indicator of functional status [11–13]. Consequently, these devices may be prescribed in clinical practice without a clear understanding of their effects. Similarly, there is insufficient guidance on optimal training durations with assistive devices or whether training time impacts their effectiveness [10–13]. Furthermore, while the general use of assistive devices is encouraged, there is limited exploration of criteria for selecting the most appropriate type of device for individuals with neurological conditions based on the specific effects of each device on gait [10–13,18]. Therefore, in clinical practice, the decision about the prescription of assistive devices indicated for individuals with neurological disorders is not based on evidence.

A systematic review evaluated the effects of the cane on the gait speed, stride length, cadence, and symmetry in individuals after stroke [19]. According to the results, no improvement in these parameters was observed with the use of the single-point and four-point cane compared to walking without cane [19]. The effects of other assistive devices, such as walkers, on the gait of these individuals, were not investigated by the authors [19]. In another systematic review, the effects of using assistive devices on individuals with PD were evaluated [20]. In this study, it was identified that, in general, canes and walkers reduced the gait speed compared to walking without device [20]. In contrast no differences were found between the use of different assistive devices [20]. However, this review included different study designs, such as case series [20], whose methodology is not strong for causal inference of an intervention [21]. Furthermore, in both reviews, the authors reported data from studies that investigated the immediate use of the devices, limiting the understanding of the effects of different training periods on the effectiveness of this intervention. Systematic reviews on the effects of using assistive devices on gait in individuals with other neurological disorders were not found.

Summarizing knowledge about the effects of using assistive devices on the gait of individuals with neurological disorders is important to guide professionals in prescribing assistive devices and evaluating their effects on these individuals. Investigating the immediate, short, and long-term effects of using these devices could provide preliminary evidence to identify important gaps in the literature and guide clinical practice. In addition, it can contribute to the design of future studies on this topic. Therefore, the aim of this systematic review is to investigate the immediate, short, and long-term effects of using conventional assistive devices (canes, crutches, and/or walkers) on gait parameters of adults with neurological disorders.

## Materials and methods

### Study protocol registration

This systematic review protocol was registered with the International Prospective Register of Systematic Reviews (PROSPERO) on 07 May 2024 (registration number: CRD42024542695) and was conducted following Preferred Reporting Items for Systematic Review and Meta-Analysis Protocols (PRISMA-P) (S1 File) [22]. The review will be reported following the Preferred Reporting Items for Systematic Review and Meta-Analysis (PRISMA 2020 statement) [23].

### Inclusion criteria for study selection

#### Type of studies.

Experimental studies will be included. Systematic reviews and qualitative studies will also be excluded, but their reference lists will be screened for relevant studies.

#### Type of participants.

We will include studies involving adult participants (age ≥ 18 years old), who had a diagnosis of neurological disorders (e.g., stroke, Alzheimer’s disease, PD, multiple sclerosis, brain and spine injuries).

#### Type of interventions.

All experimental studies using any conventional assistive device in adults with neurological disorders will be included [14]. Studies with non-conventional assistive devices such as devices with visual cues, lasers, and robotic devices will be excluded.

#### Comparisons or control.

We will select studies comparing the use of assistive devices with control group involving walking without a device, the use of assistive devices with control group with treatments withheld as placebo, sham or attention controls, and the use of different conventional assistive devices. Based on the final articles included, the results found can be grouped according to the type of control intervention.

#### Type of outcome.

The following gait parameters will be considered: speed, stride length, cadence, and distance assessed by biomechanical tools or performance-based tests.

### Data sources and search strategy

Electronic search will be carried out for articles indexed on following databases: Medical Literature Analysis and Retrieval System Online (MEDLINE Ovid), Excerpta Medica Database (Embase Classic +  Embase Ovid), Physiotherapy Evidence Database (PEDro), Scientific Electronic Library Online (Scielo), Cumulative Index to Nursing and Allied Health Literature (CINAHL Database) and Literatura Latino-Americana e do Caribe em Ciências da Saúde (LILACS). Gray literature will be searched through Web of Science and Scopus. In addition, reference lists of the included studies will be manually searched to identify additional relevant studies. The searches will not be limited by language or publication date. The search strategy includes terms related to neurological disorders, assistive devices, and gait (S2 File). The research strategy related to neurological disorders was developed based on a systematic review by Marinho-Buzelli et al. 2015 [24]. The search strategy related to assistive devices was based on three previously published reviews [19,20,25]. The search strategy related to gait was based on two previously published reviews [26,27]. The search strategy was designed according to previous studies and with the assistance of an experienced researcher.

### Selection of the studies

Initially, searches will be carried out in the databases using the designed search strategy. The search results will be saved and maintained in Rayyan Systems Inc. software [28]. Two independent reviewers (JPM, SAB) will then screen the titles and abstracts to check the eligibility criteria and remove duplicate studies. The full texts of selected articles will be evaluated by the same reviewers (JPM, SAB) and a third reviewer (CDMCF) will be forwarded to resolve any discrepancies. During the selection and screening of studies, reviewers will be blind to authors and journals. The results of the screening process will be recorded and provided in detail using an information flowchart as recommended by PRISMA.

### Data extraction

The two independent reviewers (JPM, SAB) will extract the data, which include: study details (authors, setting and year of publication), sample characteristics (type of neurological condition, inclusion and exclusion criteria, number of participants, age, sex, and clinical characteristics), methods (study design, times at which data were collected, measuring instruments and gait parameters evaluated), characteristics of interventions (assistive device used and time/frequency of use), and characteristics of comparison/control group and main results (description and effects of the intervention on the outcomes). We will extract information from the included articles and perform the meta-analysis, if possible. Any divergence will be resolved by the third reviewer (CDMCF).

### Risk of bias

The methodological quality of the included studies will be assessed using the Cochrane Risk of Bias tools [29,30]. The methodological quality will be assessed by two reviewers (JPM, SAB), and disagreements will be discussed with the third reviewer (CDMCF). We plan to assign an overall risk of bias rating as: 1) Low overall risk of bias: all domains at low risk of bias; 2) Unclear overall risk of bias: one or more domains at unclear risk of bias and no domains at high risk of bias; 3) High overall risk of bias: at least one domain at high risk of bias.

### Quality of evidence

The overall quality of the evidence will be assessed based on the Grading of Assessment, Development and Evaluation (GRADE) recommendations as follows: 1) high: more research is very unlikely to change confidence in effect estimates; 2) moderate: more research will likely have an important impact on confidence in the effect and may change the estimate; 3) low: it is very likely that more research will have an important impact on confidence in the effect and will change the estimate; and, 4) very low: any estimate of the effect is very uncertain [31].

### Dealing with missing data

We will contact the study authors directly to acquire relevant missing or unclear data. If we do not receive a response, we will explore the effects of study exclusion in a sensitivity analysis. If the effect of exclusion is significant, studies with missing or incomplete relevant data will be presented only in relation to descriptive data.

### Data syntheses

A detailed summary of results from all included studies will be provided in table and text formats. We will consider meta-analysis if there are at least two homogeneous studies (studies that investigated the effect of intervention in the same population and reported the results similarly). The meta-analysis will be performed using the Review Manager software (RevMan). The I^2^ statistic will be used to measure heterogeneity between studies. The cutoff point for significant heterogeneity will be > 50% [32]. If a minimum of 10 studies is included in the meta-analysis, reporting bias will be evaluated through a visual inspection of the funnel plot [32].

### Subgroup analysis

If sufficient comparable studies are available, subgroup analysis will be conducted in terms of the type of device used, usage time (immediate, short, and long-term), neurological condition and age of the participants.

### Sensitivity analysis

Sensitivity analyses will be performed to examine the robustness of meta-analysis. If heterogeneity is high (I^2^ >  50%), we will conduct sensitivity analyses for primary outcomes following the parameters: 1) Exclusion of studies with high risk of bias 2) Exclusion of studies with missing data, when these cannot be provided by the study authors.

## Discussion

According to our knowledge, this systematic review is the first to investigate the immediate, short, and long-term effects of using conventional assistive devices (canes, crutches, and/or walkers) on gait parameters of adults with neurological disorders. Although similar reviews were found for post-stroke and PD individuals, these reviews did not investigate the effects of all these devices or did not exclusively include experimental studies [19,20]. Additionally, these previous reviews focused on specific neurological populations [19,20]. Therefore, this review aims to summarize knowledge on this topic in a broad population, facilitating the application of current evidence in the clinical practice of caring for different neurological disorders [33].

Lee et al. (2022) investigated the effectiveness of prescribing assistive devices on gait parameters in elderly individuals [33]. According to the authors, the effects of using assistive devices on gait speed were inconsistent [33]. However, most of the studies included in this review reported data on immediate effects, not considering the potential impact of training and learning as strategies to increase device’s effectiveness [33]. Furthermore, the authors included only studies involving elderly individuals, with Parkinson’s disease, stroke, and Alzheimer’s being the only neurological disorders identified in the included studies [33]. As numerous neurological disorders affect young adult individuals, these results do not provide comprehensive insight into the effects of using these devices on the gait of individuals with neurological disorders. Moreover, since gait parameters are naturally modified by aging [34], summarizing the literature considering different age groups, as planned by this review, may provide a broader understanding of how assistive devices impact the gait of individuals with neurological conditions in different age groups.

Despite the lack of evidence regarding the effects of using assistive devices, their common use among individuals with neurological disorders is well-documented [10,15–17]. Although clinical guidelines recommend the use of these devices in populations with different neurological disorders the prescription of these devices has not been sufficiently detailed [10,12,13,35]. In the research context, studies investigating the effect of interventions on the gait of individuals with neurological disorders show inconsistency in the decision to include the use of assistive devices as an eligibility criterion for the sample of interest [36,37]. Information about the potential effects of using these devices could provide evidence to improve the design of future studies of interventions to improve gait. Considering the frequent occurrence of gait impairments in individuals with neurological disorders and the recurrent use of assistive devices in both research and clinical practice, it is essential to investigate the effects of using different devices on gait of this population.

The results of this review will provide useful information on the effects of widely used assistive devices on gait. Additionally, they will enhance the understanding of the effects of using these devices in various neurological disorders, considering a wide age range, in a comprehensive and systematic manner. Furthermore, investigation of the immediate, short, and long-term effects may generate useful information for making clinical decisions related to the training time for using these devices. If data are available to conduct a meta-analysis, the prescription of these devices can be improved based on knowledge of the effects of their use in different populations. Finally, this systematic review will highlight gaps in the literature, identifying the need for further studies focused on specific diagnostic and age populations, considering the various device types and training durations identified. These findings will contribute to guiding future research on this topic.

## Supporting information

S1 File PRISMA-P-checklist. (DOCX)

S2 File Search strategy. (DOCX)
