# Supplementary material for: Effects of using conventional assistive devices on spatiotemporal gait parameters of adults with neurological disorders: A systematic review protocol
Source: PLoS One. 2025 Apr 11;20(4):e0321019. doi: 10.1371/journal.pone.0321019 (PMC11990470; doi:10.1371/journal.pone.0321019)
Supplement: S2 File — (DOCX) [file pone.0321019.s002.docx]

**Search Strategy**

| 1 | Alzheimer Disease/ or Alzheimer.mp. |
| --- | --- |
| 2 | Dementia.mp. |
| 3 | Parkinson.mp. or Parkinson Disease/ |
| 4 | Parkinsonism |
| 5 | Movement disorder.mp. or Movement Disorders/ |
| 6 | Basal ganglia disease.mp. or Basal Ganglia Diseases/ |
| 7 | Stroke.mp. |
| 8 | Cerebrovascular accident' |
| 9 | Cerebrovascular disease |
| 10 | Cerebrovascular disorder.mp. or Cerebrovascular Disorders/ |
| 11 | Paresis.mp |
| 12 | Hemiparesis/ or hemiplegia.mp |
| 13 | Hemorrhagic Stroke or Ischemic Stroke/ |
| 14 | Spinal cord injury.mp. or Spinal Cord Injuries/ |
| 15 | Peripheral nerve injury.mp. or Peripheral Nerve Injuries/ |
| 16 | Traumatic brain injury.mp. or Brain Injuries, Traumatic/ |
| 17 | Brain Injury |
| 18 | Brain disease.mp. or Brain Diseases/ |
| 19 | Cerebellar disease.mp. or Cerebellar Diseases/ |
| 20 | Ataxia.mp |
| 21 | Dystonia.mp. |
| 22 | Huntington Chorea |
| 23 | Transverse Myelitis/ or Myelitis/ or transverse |
| 24 | Poliomyelitis.mp. |
| 25 | Autoimmune disease.mp |
| 26 | Multiple sclerosis.mp |
| 27 | Demyelinating Disease/ |
| 28 | Guillain‐Barre syndrome.mp. |
| 29 | Charcot‐Marie‐Tooth.mp |
| 30 | Nervous System Diseases.mp. / |
| 31 | Neurological conditions |
| 32 | Neurological diseases |
| 33 | Neurological disorders |
| 34 | Neuromuscular disease.mp. or Neuromuscular Diseases/ |
| 35 | Assistive Device |
| 36 | Canes |
| 37 | Crutches |
| 38 | Gait aid |
| 39 | Gutter frame |
| 40 | Mobility Aid |
| 41 | Mobility Assistive Device |
| 42 | Mobility device |
| 43 | Rollator |
| 44 | Self-help Devices |
| 45 | Stick |
| 46 | Walkers |
| 47 | Walking Aids |
| 48 | Walking Device |
| 49 | Walking frame |
| 50 | Walking stick* |
| 51 | Ambulatory |
| 52 | Gait |
| 53 | Locomotion |
| 54 | Mobility |
| 55 | Walk |
| 56 | Walking |
| 57 | 1 or 2 or 3 or 4 or 5 or 6 or 7 or 8 or 9 or 10 or 11 or 12 or 13 or 14 or 15 or 16 or 17 or 18 or 19 or 20 or 21 or 22 or 23 or 24 or 25 or 26 or 27 or 28 or 29 or 30 or 31 or 32 or 33 or 34 |
| 58 | 35 or 36 or 37 or 38 or 39 or 40 or 41 or 42 or 43 or 44 or 45 or 46 or 47 or 48 or 49 or 50 |
| 59 | 51 or 52 or 53 or 54 or 55 or 56 |
| 60 | 57 and 58 and 59 |
